# Sesamin Attenuates VEGFA-Induced Angiogenesis via Inhibition of Src and FAK Signaling in Chick Chorioallantoic Membrane Model and Human Endothelial EA.hy926 Cells

**DOI:** 10.3390/biomedicines11010188

**Published:** 2023-01-11

**Authors:** Tanyaporn Keratibumrungpong, Warunee Srisuthtayanont, Orawan Wanachewin, Jeerawan Klangjorhor, Thanyaluck Phitak, Peraphan Pothacharoen, Thuzar Hla Shwe, Prachya Kongtawelert

**Affiliations:** Thailand Excellence Center for Tissue Engineering and Stem Cells, Department of Biochemistry, Faculty of Medicine, Chiang Mai University, Chiang Mai 50200, Thailand

**Keywords:** angiogenesis, sesamin, VEGF, EA.hy926, NOTCH1

## Abstract

Sesamin, a major phytochemical in sesame seeds and oil, has been reported to have effects on physiological and pathological angiogenesis in several studies. Nevertheless, the underlying mechanisms of sesamin’s effect on angiogenesis are not understood well enough. This study aimed to investigate its effect on both physiological and pathological angiogenesis using the in vivo chick chorioallantoic membrane (CAM) model and the in vitro human endothelial cell line, EA.hy926, model. Sesamin inhibited the VEGFA-induced pathological angiogenesis significantly, although no effect was seen on angiogenesis without induction. It reduced the formation of vascular branches in the VEGFA-treated CAMs and also the proliferation and migration of EA.hy926 endothelial cells induced by VEGFA. Sesamin impeded the VEGF-mediated activation of Src and FAK signaling proteins, which may be responsible for sesamin-mediated reduction of pathological angiogenesis. Moreover, the effect of sesamin on the expressions of angiogenesis-related genes was then investigated and it was found that both mRNA and protein expressions of Notch1, the key pathway in vascular development, induced by VEGFA, were significantly reduced by sesamin. Our results altogether suggested that sesamin, by inhibiting pathological angiogenesis, has the potential to be employed in the prevention or treatment of diseases with over-angiogenesis, such as cancers.

## 1. Introduction

Angiogenesis is the process of developing new vessels from the pre-existing vasculature and it occurs throughout the entire lifespan of an organism under both physiological and pathological conditions [1]. Since the vascular network is mostly quiescent in adulthood, physiological angiogenesis mainly occurs in developmental contexts such as embryonic blood vessels [2] and endochondral bone formation [3]; the exception is the female reproductive tract such as cycling ovary and uterus during pregnancy, where the formation of new vessels is required [4]. However, in numerous pathological conditions such as tumors or inflammation, the resting vasculature can be reactivated [5]. 

Angiogenesis takes place in a number of sequential steps: endothelial cell activation, cell sprouting, proliferation, migration, lumen formation, cell remodeling, and maturation. It is initiated in normally quiescent endothelial cells (ECs) upon receiving pro-angiogenic factors such as Vascular Endothelial Growth Factor (VEGF), the principal angiogenic factor. When VEGF contacts the VEGF receptor (VEGFR), the receptor generates the signals to develop a vascular system in the embryo or to generate blood vessels and lymphatic vessels in adults [6]. Among various VEGFs (VEGFA, VEGFB, VEGFC, and VEGFD) and VEGFRs (VEGFR1, VEGFR2, and VEGFR3), VEGFA/VEGFR2 signaling prominently mediates angiogenesis processes of blood vessels. Diverse downstream signaling pathways including MAPK pathways: p38, ERK, phospholipase γ (PLCγ), Src, and FAK pathways are reported to regulate the cell survival, proliferation, migration, and cellular attachment of endothelial cells [7,8]. 

VEGF/VEGFR signaling also has an influence on a variety of other pathways. Notch signaling is an evolutionally conserved pathway involved in the determination of fate of many tissues and cell types including endothelial cells. Notch receptors and their ligand, the delta-like ligand 4 (Dll4), are responsible for tip and stalk cell differentiation from endothelial cells (ECs) in nascent vascular sprouts, while the migration and proliferation of tip and stalk cells governs the formation, elongation, and maturation of new vessels eventually [5,9]. 

As angiogenesis is regulated by the balance between pro-angiogenic and anti-angiogenic signals, any disturbance in that balance would lead to pathological conditions. While an insufficient vascular supply results in ischemic diseases, excess neovascularization is a hallmark of solid tumors [10,11]. The hypoxic and acidic nature of the tumor microenvironment (TME) induces the secretion of VEGFs from cells such as macrophage, fibroblasts, endothelial cells, and tumor cells, rendering TME to be angiogenic, which in turn enhances the tumor growth and metastasis [12]. Accordingly, the inhibition of angiogenesis has been one of the therapeutic options in cancer and there are a variety of approved anti-angiogenic drugs; Bevacizumab, a neutralizing monoclonal antibody directed against VEGF, and tyrosine kinase inhibitors (RTKi) such as sorafenib, sunitinib, cediranib, and axitinib are the most commonly used ones in this category [13,14]. Moreover, the monoclonal antibody against Dll4 also exhibited inhibitory properties on breast tumor growth [15]. 

Sesamin (*Sesamun indicum* L.), a lignan found in sesame seeds and oil, is reported to have biological effects including chemoprevention, anti-inflammation, and anti-oxidant properties [16,17,18]. The effect of sesamin on angiogenesis under physiological and pathological conditions has been studied by different scientists, however, the findings were contradicting and confusing. A study has shown that sesamin increases the in vitro and in vivo angiogenic processes including endothelial cell proliferation, migration, tube formation, and neovascularization via activation of signaling modulators: ERK, Akt, focal adhesion kinase (FAK) and p38 mitogen-activated protein kinase (MAPK) [19]. In contrast, sesamin was shown to inhibit angiogenesis in other studies [20,21]. In addition to these controversial findings for the effect of sesamin on angiogenesis, the underlying signaling mechanisms and its effect on Notch signaling have not yet been elucidated enough. 

This study thus aimed to investigate the effect of sesamin on angiogenesis in an in vivo model using the chick chorioallantoic membrane (CAM) [22] and in EA.hy926, a human endothelial cell line. Although sesamin did not affect either in vitro or in vivo physiological angiogenesis, it could inhibit VEGFA-induced pathological angiogenesis in the CAM model. This might be due to its ability to decrease endothelial cells proliferation and migration via inactivation of Src and FAK signaling. Moreover, inactivation of those pathways by sesamin might also result in a lower expression of NOTCH, eventually inhibiting Dll4-Notch signaling.

## 2. Materials and Methods

### 2.1. Chemicals

Methylthiazoletetrazolium (MTT) (PubChem CID: 64965), Dimethyl sulfoxide (DMSO) (PubChem CID: 679), and Resazurin (PubChem CID: 11077) were obtained from Sigma Chemical, Inc. (St. Louis, MO, USA). Recombinant human vascular endothelial growth factor A (Srf21-derived, Catalog number 293-VE) was purchased from R&D systems^®^ (Minneapolis, MN, USA). Illustra RNAspin Mini RNA Isolation Kit and the primers for real-time RT-PCR were acquired from GE Healthcare Europe GmbH (Freiburg, Germany) and BioDesign (Bangkok, Thailand), respectively. A Tetro cDNA Synthesis kit and a SensiFAST™ SYBR^®^ No-ROX Kit were purchased from BIOLINE (London, UK). Angiogenesis Antibody Sampler Kit, Notch1 (D1E11) and β-Actin Rabbit monoclonal antibody were procured from Cell Signaling Technology^®^ (Beverly, MA, USA).

### 2.2. Sesamin Preparation

Sesamin seeds were procured from the Lampang province of Thailand, and the voucher specimens (BKF no. 138181) were approved by the National Park, Wildlife and Plant Conservation Department, Ministry of Natural Resources and Environment, Bangkok, Thailand. Sesamin was extracted from the seeds by using the method reported in the previous study [23]. The sesamin obtained was first dissolved in DMSO to prepare the stock solution (100 mM) before being diluted with the culture media to the required concentrations. All the experiments were carried out in compliance with the relevant guidelines of the institution. 

### 2.3. In Vivo Angiogenesis Model: Chick Chorioallantoic Membrane (CAM) Assay

Fertilized chicken eggs were incubated at 37 °C and approximately 50–60% humidity. On day 3 of incubation, 5–6 mL of albumen was aspirated to detach the developing CAM from the top part of the shell. On day 8, a window of around 1.5 cm^2^ was gently opened on the wide end of the egg without damaging the embryo. A plastic ring was placed directly on the top of the CAM. Various concentrations of sesamin and/or 20 ng/mL of VEGFA were added directly onto the plastic rings. The eggs were transferred back into the incubator and the numbers of vascular branches were counted at day 10 of incubation by photographing the CAM area of each egg. Images were analyzed by counting the number of branching vessels in the plastic ring. Scores of primary-, secondary-, tertiary-, and quaternary-branching vessels are denoted by 1, 2, 3, and 4, respectively. Further branching vessels with more than quaternary-branching were scored as 5. Scores from each group were averaged by the number of eggs and the average score of each group was normalized to that of the control group [22,24].

### 2.4. Cell Line and Culture

EA.hy926, a human umbilical vein endothelial cell line, was purchased from ATCC^®^ (CRL2922™). This cell line was derived by fusing human umbilical vein endothelial cells with the permanent human cell line, A549. This study used EA.hy926 because this cell line shows endothelial characteristics and more cells can be obtained than from the primary cells [25]. The cells were cultured as a confluent monolayer in Dulbecco’s Modified Eagle’s Medium (DMEM), containing 10% fetal bovine serum and 2% HAT (100 μM hypoxanthine, 0.4 μM aminopterin, and 16 μM thymidine). Cells were maintained in a humidified incubator with 5% CO_2_ at 37 °C. A cell passage of six or seven was used in the experiments.

### 2.5. MTT Assay

To determine the toxicity on cells and select the optimal concentrations of sesamin, a MTT assay was performed. EA.hy926 cells were placed in a 96-well-plate (1000 cell/well) and incubated overnight. After cells were treated with various sesamin concentrations for 24 h, the culture media were discarded and replaced with 100 μL of MTT (0.5 mg/mL) solution for 4 h. Then, the MTT agent was discarded and 100 μL dimethyl sulfoxide (DMSO) was added into each well to solubilize the formazane crystals. The absorbance was measured at 540 nm using a microplate reader and the percentage cell survival compared to the controls was calculated as follows:

(1) $$\mathrm{Percentage}\;\mathrm{of}\;\mathrm{survival}=\;\frac{\left(\mathrm{OD}\;\mathrm{of}\;\mathrm{sample}\;\times \;100\right)}{\left(\mathrm{OD}\;\mathrm{of}\;\mathrm{DMEM}\;\mathrm{control}\right)}$$

### 2.6. In Vitro Wound Healing Assay

To determine the migration ability of EA.hy926 cells, an in vitro wound healing assay was performed. EA.hy926 cells were seeded into 24 well plates (200,000 cells/well) and incubated. After 24 h of incubation, a line was scraped through the monolayer of the cells using a 250 μL-pipette tip (wounding). The cells were then washed with PBS and treated with various concentrations of sesamin and/or 20 ng/mL VEGFA. The wounds were photographed at different time points under a light microscope (40× magnification). The measurements of the scratched region were calculated using AxioVision Analytic Software version 4.7 from Carl Zeiss (Jena, Germany). [20] The migration ability was calculated as follows:

(2) $$\mathrm{Migration}\;\mathrm{ability}=100-\left[\frac{(\mathrm{Width}\;\times \;\mathrm{hrs})\;\times \;100}{\left(\mathrm{Width}\;0\;h\right)}\right]$$

### 2.7. AlamarBlue Assay

The extent of cell proliferation was assessed using the AlamarBlue assay. EA.hy926 cells were seeded into 96 well plates (1000 cells/well) and incubated at 37 °C, 5% CO_2_ for 24 h. The culture media was then discarded, and the cells were treated with various concentrations of sesamin and/or 20 ng/mL VEGFA for 1 week. Every 24 h, the culture media was discarded and replaced with 10% (*v*/*v*) Alarmar Blue fluorescent dye in media for 4 h at 37 °C. The absorbance of the media was read at 540 and 620 nm using a microplate reader spectrophotometer. The absorbance was measured at wavelengths of 540 and 620 nm. The percentages of the differences in reduction were calculated, and the data represented as percentages of the differences in reduction relative to those of the control group.

### 2.8. Gene Expression by Real-Time Reverse Transcription-Polymerase Chain Reaction (RT-PCR) 

Real time RT-PCR was used to examine the gene expression in EA.hy926 in response to treatment with various concentrations of sesamin and/or 20 ng/mL VEGFA. After 24- or 48-h incubation periods, cells were lysed, and the total RNA was isolated using the RNA extraction kit (GE Healthcare) following the manufacturer’s protocol. Samples were treated with DNase before washing and elution steps. After extraction, the amount of total RNA in samples was measured using nanodrop spectrophotometer (Thermo Scientific, Waltham, MA, USA), and 200 ng of total RNA were converted to cDNA using a Tetro cDN A Synthesis Kit in a final volume of 20 μL. Reaction conditions were set as suggested by the manufacturer. After reverse-transcription, the product cDNA was diluted with RNase free water in 1:10 dilution. Then, 8 μL of the cDNA mixture was used for real-time PCR experiments. Real time PCR was performed using a SensiFastTM SYBR^®^ No-ROX kit on Chromo4TM Four-Color Real-Time Detector from Bio-Rad (Hercules, CA, USA) in a final volume of 20 μL. The primer concentration was 500 nM. Forty cycles of PCR amplification were performed at 95 °C for 5 s and 60 °C for 10 s. The primers used are shown in Table 1. The relative expression for each gene was normalized to that of GAPDH and against the control group by the 2^ΔΔCT^ method [26]. Additional information on normalized gene expression (ΔCт) of each target gene against GAPDH gene was described in Supplementary Materials.

### 2.9. Western Blotting Analysis

EA.hy926 cells were treated with various sesamin concentrations and/or VEGFA and incubated at 37 °C, 5% CO_2_. At the indicated times, the cells were lysed with lysis buffer containing 50 mM Tris-HCL, pH7.4, 250 mM NaCl, 0.5% NP-40, 5 mM EDTA, and 50 mM NaF along with protease and phosphatase inhibitors (Roche Diagnostics GmbH, Mannheim, Germany). An equal volume of whole cell lysate was electrophoresed and transferred to a nitrocellulose membrane. After blocking with 5% skim milk in 0.05% PBS-TWEEN, the membranes were incubated with specific primary antibodies. After the incubation of the secondary antibodies and washing, specific protein bands were developed using Supersignal West Femto Substrate (Thermo Scientific, Rockford, IL, USA) and were photographed using the molecular chemidoc XRS system (Bio-Rad, Hercules, CA, USA). The band density was analyzed using TotalLab TL120 software version 2006 and calculated in relation to the control sample. The beta actin was used as an internal protein control.

### 2.10. Statistical Analysis

All data are given as mean ± standard error of mean (SEM) from triplicate samples of three or two independent experiments. One-way analysis of variance (one-way ANOVA) and student’s t-test were used to compare the treatment and control conditions using data from three or two independent experiments, respectively. Statistical significance was assumed at *p* < 0.05.

## 3. Results

### 3.1. The Effect of Sesamin on Angiogenesis in In Vivo Chick Chorioallantoic Membrane (CAM) Model

**Figure 1:**
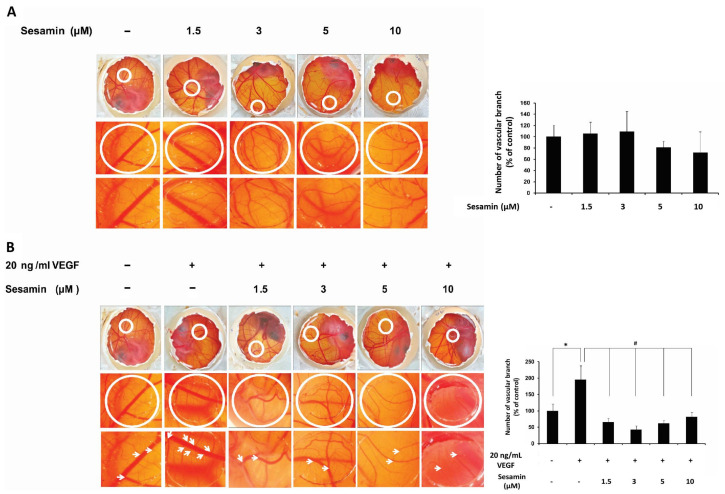
The effect of sesamin on angiogenesis in the chick chorioallantoic membrane (CAM) model under physiological and pathological conditions. (**A**) Patterns of vascular branching and its quantification in uninduced CAM treated with various sesamin concentrations. (**B**) Patterns of vascular branching and its quantification in CAM cotreated with various sesamin concentrations and 20 ng/mL VEGF. Fertilized chicken eggs were incubated for 8 days. After opening the eggs shield, a plastic ring was plated on the chick chorioallantoic membrane, and the treatments in the volume of 20 μL were added in the ring. For each condition, three eggs were used. Two days later, the number of vascular branches was quantified as described in Materials and Methods and the percentage of vascular branching in treatment conditions compared to the control was calculated. The uppermost rows in the figures show the top view of the experimental eggs, where the circle indicates the treatment area. The middle and lower rows show the treatment areas of each condition under a stereo microscope with 25× magnification and 45× magnification, respectively. The white arrows show the vascular branching points counted. The data are expressed as the mean ± SEM. The results are representative of two independent experiments. * indicates a significant difference compared with the untreated control at *p*-value less than 0.05. # indicates a significant difference compared with VEGFA treatment at *p*-value less than 0.05.

This experiment was preliminary to the investigation of the effect of sesamin on both physiological and pathological angiogenesis in an in vivo model. The experiment focused on the number of vascular branches formed on the CAM. The results showed that various concentrations of sesamin (1.5, 3.0, 5.0, and 10 μM) did not cause significant changes on the formation of new branching vessels in the CAM. Therefore, the concentrations of sesamin used did not affect in vivo angiogenesis under physiological conditions (Figure 1A). 

To mimic the pathological conditions with reported increased angiogenesis, such as cancer, the CAM was treated with the vascular endothelial growth factor A (VEGFA) [22]. After 48 h treatment of 20 ng/mL VEGFA, the number of branches of capillaries developed from pre-existing vessels in the CAM had increased compared to the untreated control. However, co-treatment with sesamin significantly decreased the number of vascular branches induced by VEGFA in the CAM (Figure 1B). The results indicated that sesamin could inhibit the angiogenic effect of VEGFA, prompting the therapeutic potential of sesamin in pathological conditions with increased angiogenesis such as cancer. Therefore, the effects of sesamin on angiogenesis were further investigated in the endothelial cell line EA.hy926. 

### 3.2. The Effect of Sesamin on Cytotoxicity and Proliferation of Human Endothelial Cell Line EA.hy926

**Figure 2:**
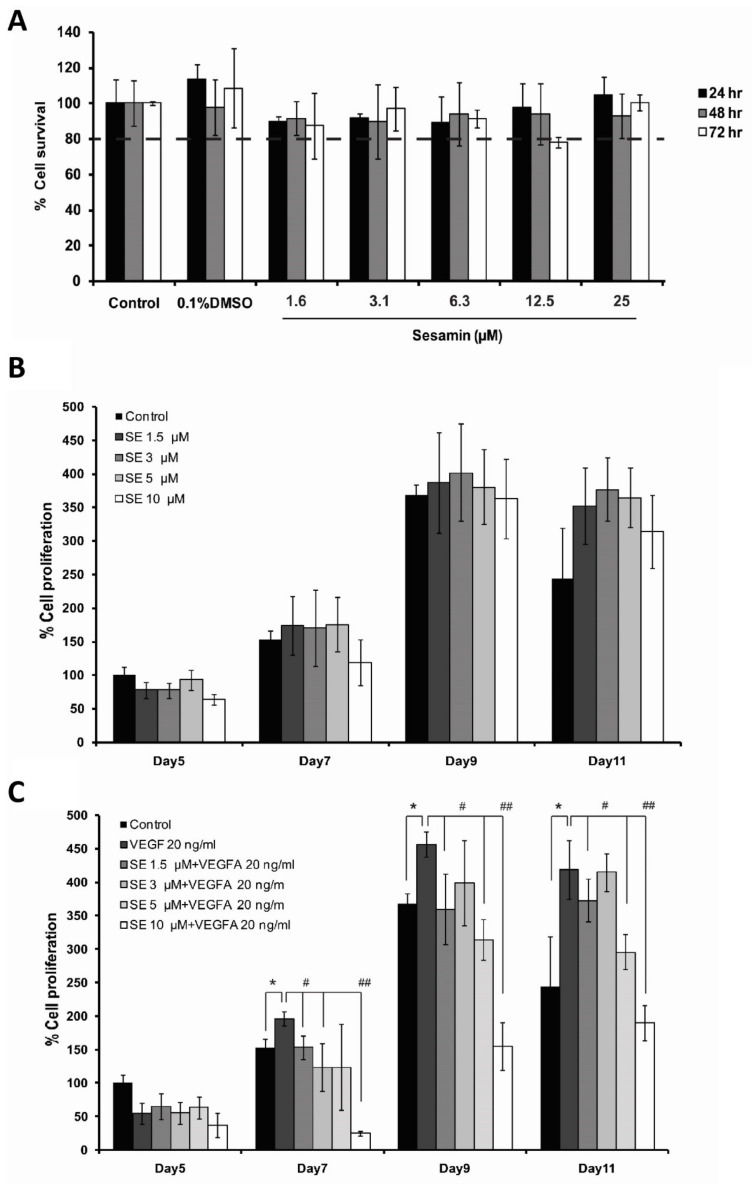
The effect of sesamin on cytotoxicity and proliferation of human endothelial cell line, EA.hy926. (**A**) The cytotoxic effect of various doses of sesamin on EA.hy926 for 24, 48, and 72 h was investigated using the cell viability (MTT) assay. (**B**,**C**) The effect of sesamin (1.5–10 μM) on the proliferation of the EA.hy926 cells was investigated using the AlamarBlue assay (**B**) in the absence of VEGFA and (**C**) in the presence of VEGFA (20 ng/mL). The data are expressed as the mean ± SEM from three independent experiments. * indicates a significant difference compared with untreated control at *p*-value less than 0.05. # and ## indicates significant differences compared with VEGFA treatment at *p*-values less than 0.05 and 0.01, respectively.

Before the effects of sesamin on angiogenesis could be determined in the in vitro model, its cytotoxicity on the cell line was first examined. EA.hy926, a human endothelial cell line, was treated with sesamin, 1.6 to 25 μM concentrations, for 24, 48, and 72 h and the percentage of cell viability compared to the untreated control was determined by the MTT assay. The percentages of cell viability under all conditions were greater than 80% relative to the control, indicating that sesamin up to 25 μM had no significant cytotoxic effect on EA.hy926 (Figure 2A). DMSO, in which sesamin was solubilized, also showed no effect on the cell viability at the concentration of 0.1%. According to the result, sesamin at concentrations of 1.5, 3.0, 5.0, and 10 μM were used in further experiments.

Since proliferation of endothelial cells is an important step in angiogenesis supporting the elongation of new blood vessels, the effect of sesamin on endothelial cell proliferation was investigated. EA.hy926, human endothelial cells, were treated with various concentrations of sesamin (1.5–10 μM), with or without VEGFA, and the cell proliferation was analyzed using the AlamarBlue assay at days 5, 7, 9, and 11. The percentage of proliferation of uninduced EA.hy926 cells was not significantly affected by the treatment with sesamin, compared with that of the control, indicating that sesamin has no effect on angiogenesis itself (Figure 2B). 

VEGFA expression is high in various pathological conditions such as cancer and inflammation. Moreover, previous studies have reported that in vivo induction with a single VEGFA treatment, VEGFA (164/5), can result in the formation of new blood vessels that differ from normal vessels [27]. Therefore, pathological angiogenesis under these conditions were simulated by induction of the endothelial cells with a high concentration of VEGFA. When EA.hy926 cells were induced with 20 ng/mL VEGFA, the percent proliferation of EA.hy926 compared to the control was significantly increased, starting from day 7. Here, cotreatment with various concentrations of sesamin reduced the enhanced proliferation of EA.hy926 significantly. Sesamin at a concentration as low as 1.5 μM can significantly inhibit the induced proliferation of the cells by 25%, 22%, and 11% at day 7, 9, and 11, respectively (Figure 2C). The result reflects the role of sesamin in the inhibition of angiogenesis under pathological conditions through the reduction of endothelial cell proliferation. 

### 3.3. The Effect of Sesamin on Endothelial Cell Migration under Physiological and Pathological Conditions

**Figure 3:**
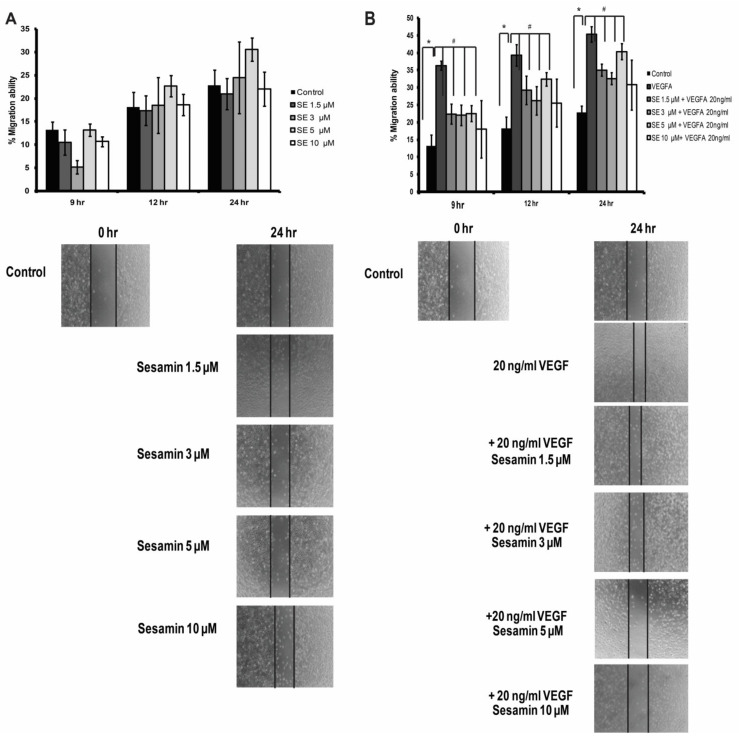
The effect of sesamin on the migration ability of human endothelial cell line, EA.hy926, under physiological conditions and pathological conditions. (**A**) The effect of sesamin (1.5–10 μM) on the migration ability, represented by the wound area, in the uninduced EA.hy926 cells. (**B**) The effect of sesamin (1.5–10 μM) on the migration ability, represented by the wound area, in the EA.hy926 cells induced with 20 ng/mL VEGFA. The monolayer of EA.hy926 cells were scratched with a 250 μL-pipette tip (wounded) and treated (at 0 h of treatment) with sesamin and/or VEGFA for 24 h. At the indicated time points (9, 12, and 24 h), the cells were photographed using a light microscope with 40× magnification. The dermacation line between the wound area and the area with packed cells was indicated by the black line. The enclosed wound area between the two black lines was measured as a representative for the rate of migration. The data are expressed as the mean ± SEM from three independent experiments. * indicates a significant difference compared with the untreated control at *p*-value less than 0.05. # indicates a significant difference compared with the VEGFA treatment at *p*-value less than 0.05.

During angiogenesis process, endothelial cells migrate through the basement membrane, leading to the generation of new sprouting vessels. As the migration process is another crucial process in angiogenesis, the effect of sesamin on the migration of endothelial cells was also investigated in this study. After the monolayer of human endothelial cells, EA.hy926, was wounded using a 250 μL-pipette tip, they were treated with various concentrations of sesamin for 9, 12, and 24 h. After each incubation-time point, the size of the wound gap was calculated with Axio Vision analytic software to examine the migration ability of the cells. In un-induced EA.hy926, the migration ability was not changed significantly by the treatment with various concentrations of sesamin (1.5–10 μM) (Figure 3A). This result showed that sesamin did not affect the migration process of the endothelial cells under the physiological condition. For the monolayer of endothelial cells cotreated with 20 ng/mL VEGFA and sesamin, VEGFA significantly induced the migration ability of EA.hy926 as the wound space inflicted was reduced obviously after 9 h (Figure 3B). However, co-treatment with sesamin obviously diminished the VEGFA induced-migration in EA.hy926 cells at all three incubation periods; the percentages of migration ability compared to the control was 45% in VEGFA treated EA.hy926 cells and it was reduced to only 35% in cells co-treated with VEGFA and sesamin, as low as 1.5 μM, at 24 h-time point (Figure 3B). These data indicated that sesamin suppressed the migration of endothelial cells induced under the influence of VEGFA, further confirming that sesamin can inhibit angiogenesis in pathological conditions although it has no effect on the angiogenesis without induction. 

### 3.4. The Effect of Sesamin on Activation of VEGFA Signaling Pathways in Human Endothelial Cells EA.hy926

Following the pre-treatment with sesamin, EA.hy926 cells were induced with VEGFA to study the effect of sesamin on the activation status of signaling pathways such as ERK, p38, PLCγ1, Src, and FAK, which are reported to be responsible for the proliferation and/or migration of endothelial cells induced by VEGFA [7].

**Figure 4:**
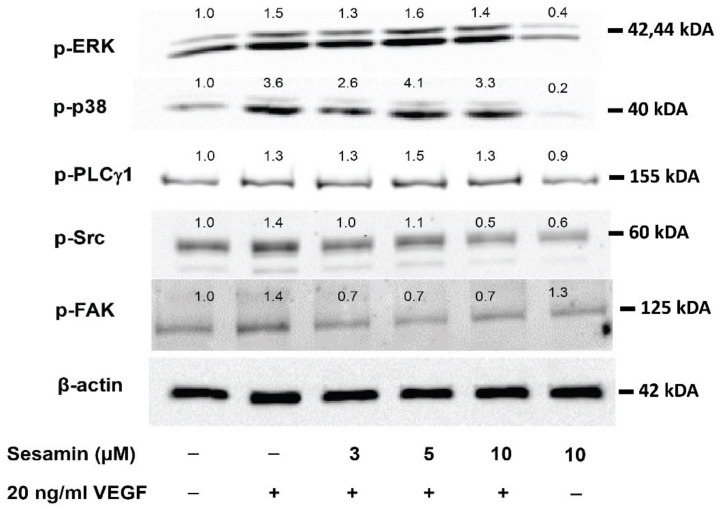
The effect of sesamin on the activation of intracellular signaling pathways induced by VEGFA in human endothelial cell line, EA.hy926. Following the pretreatment with sesamin (3–10 μM) for 2 h, EA.hy926 cells were treated with 20 ng/mL VEGFA for 5 min to study the expression of phosphorylated ERK, p38, and PLCγ1 or for 30 min to study that of phosphorylated Src and FAK. The expression levels of the phosphorylated forms were determined by western blot analysis using their specific antibodies. β actin was used as a loading control. Each sample was prepared for the detection of all signaling proteins together. The number above each band shows band density relative to its control.

The results of the western blot assay showed that VEGFA (20 ng/mL) activated all signaling pathways; the expression of phosphorylated forms of ERK, p38, PLCγ1, Src, and FAK proteins were obviously enhanced by VEGFA treatment in EA.hy926 cells. Interestingly, pre-treatment with sesamin (3, 5, and 10 μM) can diminish the expression of phosphorylated Src and FAK proteins augmented by VEGFA, although it had no effect on the VEGFA-induced expression of phosphorylated ERK, p38, and PLCγ1. Moreover, 10 μM sesamin alone slightly reduced the phosphorylation of ERK, p38, and Src compared to the untreated control, but had no effect on that of PLCγ1 and FAK (Figure 4). Taken together, it was suggested that sesamin decreased VEGFA-induced proliferation and migration of EA.hy926 via inhibition of activation of Src and FAK.

### 3.5. The Effect of Sesamin on Angiogenic Gene Expression in Human Endothelial Cells EA.hy926

It is well known that VEGFA signaling has effects on the expression of the genes related to angiogenesis [5,27]. Since sesamin could interfere with some of the intracellular signaling induced by VEGFA in EA.hy926, it was expected to have some impacts on the expression of these genes. This experiment investigated the expression of angiogenic genes, namely NOTCH1 receptor 1 (*NOTCH1*), delta like 4 (*Dll4*), vascular endothelial growth factor A (*VEGFA*), Kinase Insert Domain Receptor or VEGF receptor 2 (*KDR*), Angiopoietin-1 (*ANG1*), Angiopoietin-2 (*ANG2*), and Tie 2 receptor (*Tie2*), using real time RT-PCR. 

**Figure 5:**
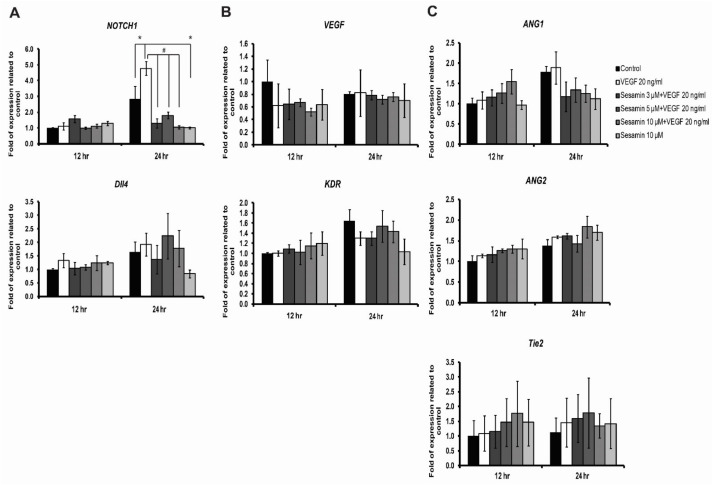
The effect of sesamin on (**A**) NOTCH1 and Dll4 gene expression, (**B**) VEGFA and KDR gene expression and (**C**) ANG1, ANG2, and Tie2 gene expression in human endothelial cell line, EA.hy926. Cells were treated with 20 ng/mL VEGFA alone or cotreated with VEGFA and various concentrations of sesamin (3, 5, and 10 μM) for 12 and 24 h and the relative mRNA expression of the specific genes compared to that of GAPDH was determined by real time RT-PCR. The data are expressed as the mean ± SEM from three independent experiments with technical triplicates in each. * indicates a significant difference compared with untreated control at *p*-value less than 0.05. # indicates a significant difference compared with VEGFA treatment at *p*-value less than 0.05.

After EA.hy926 cells were incubated with VEGFA (20 ng/mL) alone or together with various concentrations of sesamin (3, 5, and 10 μM) for 12 h and 24 h, the abovementioned genes were examined. For genes involved with specifying tip and stalk cells, VEGFA induced the expression of *NOTCH1* significantly after 24 h of incubation, but not that of *VEGF* or *Dll4* gene. In case of the VEGFA receptor, the *KDR* gene, VEGFA decreased the expression, but not to a significant extent (Figure 5A,B). Here, sesamin at various concentrations was found to significantly reduce the induction of *NOTCH1* mediated by VEGFA treatment. However, no significant influence of sesamin was seen on the expression of other genes, *Dll4, VEGF,* or *KDR*, induced by VEGFA (Figure 5A,B).

Regarding the expression of genes controlling the interaction between endothelial and mural cells, thereby regulating the stabilization of the vasculature [5], neither of the expressions of *ANG1*, *ANG2* nor that of their receptor *Tie2* were changed significantly by any of the treatments, either by VEGFA alone or cotreatment of VEGFA and sesamin (Figure 5C). Although the expression of *ANG2,* which is required for the initiation of the neovascularization, was increased after VEGFA treatment, the extent was not enough to be significant. Interestingly, cotreatment of sesamin with VEGFA, in a 12-h incubation period, can increase the expression of *ANG1,* which is responsible for the recruitment of mural cells stabilizing the newly formed vessels, although it was not significant. However, this effect disappeared in the 24-h incubation period (Figure 5C). 

These data speculated that sesamin hindered the induction of angiogenesis through the reduction of the VEGFA-induced *NOTCH1* expression. This speculation was confirmed by investigation of the NOTCH1 protein expression in the following experiment. 

### 3.6. The Effect of Sesamin on NOTCH1 Protein Expression in the Endothelial Cell Line EA.hy926

The NOTCH receptor 1 (NOTCH1) plays an important role in the sprouting step of angiogenesis. The balance of NOTCH1 activation status between tip cells and stalk cells determines the formation and branching of new vessels [5,28]. 

**Figure 6:**
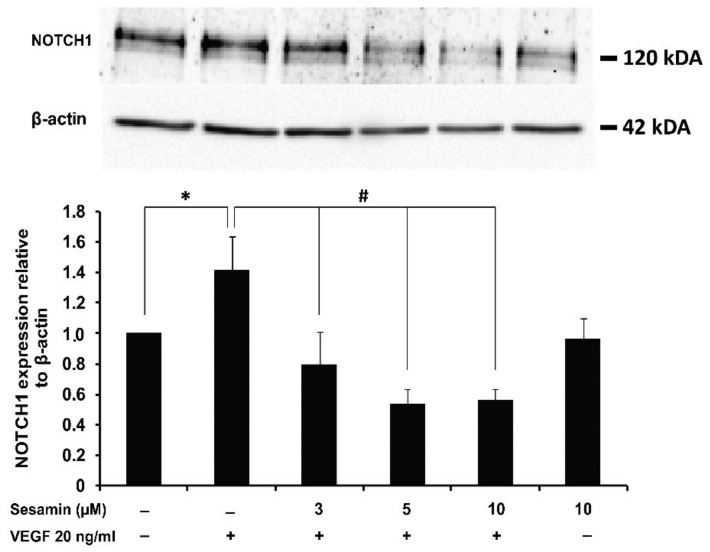
The effect of sesamin on NOTCH1 expression in human endothelial cell line, EA.hy926. Cells were treated with 20 ng/mL VEGFA alone or cotreated with VEGFA and with various concentrations of sesamin (3, 5, and 10 μM) for 24 h. The relative NOTCH1 protein expression compared to the control was examined by western blot analysis and the band intensity was normalized to the corresponding β-actin. The data are expressed as the mean ± SEM from three independent experiments. * indicates a significant difference compared with the untreated control at *p*-value less than 0.05. # indicates a significant difference compared with the VEGFA treatment at *p*-value less than 0.05.

This experiment investigated the effect of sesamin on NOTCH1 expression in EA.hy926 using western blotting. After EA.hy926 cells were treated with 20 ng/mL of VEGFA for 24 h, the expression of NOTCH1 protein was increased and cotreatment with sesamin inhibited this induction significantly, corresponding to the result of mRNA analysis in the previous experiment (Figure 6). These results further confirmed the suggestion that sesamin prevents the angiogenesis induced by VEGFA through the reduction of both mRNA and protein expression of NOTCH1, eventually reducing the proliferation and migration of EA.hy926 cells.

## 4. Discussion

The angiogenesis process occurs throughout the entire life of vertebrates and plays an important role in both the physiological and pathological conditions [5,6]. Abnormal angiogenesis, either insufficient or excess, may creating implications in a variety of diseases such as placental insufficiency, ischemic heart disease, or tumor growth [11]. Having an increased vascular supply is essential for tumors not only to match with its high demand of nutrients but also to enhance its dissemination and metastasis [10]. Consequently, VEGF signaling inhibitors have been used as one of the therapeutic options in cancer [13]. Many phytochemicals have also been investigated for their effect on angiogenesis; previous studies have reported the effect of the phytochemical sesamin (*Sesamun indicum* L.), with known anti-inflammatory and anti-oxidant activities on the angiogenesis process. Nevertheless, their findings were contradicting as Tsai et al. reported sesamin as being anti-angiogenic while Chung et al. stated that sesamin induced angiogenesis [19,20]. Moreover, the effect of sesamin on the NOTCH signaling pathway, the key signaling in different stages of angiogenesis, has not yet been elucidated. Hence, this study attempted to confirm the effect of sesamin on angiogenesis and the underlying signaling pathways in both in vitro and in vivo using the human endothelial cell line, EA.hy926, and the chick chorioallantoic membrane (CAM) models, respectively. 

We first investigated the effect of sesamin on in vivo angiogenesis in the CAM model and the formation of vascular branches in CAMs was not affected by sesamin, reflecting its lack of influence on the physiological angiogenesis. This finding is contrary to that of Chung et al., in which sesamin significantly increased neo-vascularization using the Matrigel model in male BALB/c mice [14]. It appears that different in vivo methods used to observe the formation of vascular branching may yield different results. We then examined the effect of sesamin on angiogenesis under pathological conditions using vascular endothelial growth factor A (VEGFA) as an instigator in the same CAM model. VEGF, an inflammatory cytokine, is the key driver of sprouting angiogenesis and is well-known to be overexpressed in tumor cells, via induction of HIF-1α under hypoxic conditions, promoting proliferation and migration of vascular endothelial cells and blood vessels outgrowth, thereby assisting the growth and metastasis of tumors [10,29]. Hence, we treated the CAM with VEGFA, the major VEGF involved in the growth of blood vessels, to mimic the pathological conditions of tumors, and it was found that VEGFA doubled the vascular branches formation in the CAM. This effect of angiogenesis induction by VEGFA was significantly reduced by cotreatment with sesamin, prompting the role of sesamin in the inhibition of pathological angiogenesis mediated by VEGF. 

Next, we further studied the effect of sesamin on angiogenesis and its underlying mechanisms, including its effect on NOTCH signaling, using the in vitro cell model, human endothelial cells EA.hy926. During the sprouting process of a new vessel, quiescent endothelial cells become activated and secrete enzymes that degrade extracellular matrix (ECM) proteins in basement membranes, allowing endothelial cells to migrate through. Then, endothelial cells in a nascent sprout are differentiated into two cell types, namely tip cells and stalk cells: tip cells are highly migratory and responsible for sensing the directional cues, thereby defining the route of the new sprouts and stalk cells, which highly proliferate and support sprout elongation [9]. Hence, the effect of sesamin on the proliferation and migration of endothelial cells was studied in EA.hy926 endothelial cells. VEGFA indeed induced the proliferation and migration of EA.hy926 as expected, in accordance with the findings of previous studies in endothelial cells, HUVEC and EA.hy926 [19,30,31]. Sesamin was found to inhibit the induced proliferation and migration significantly, although it had no significant influence on the uninduced EA.hy926 cells. Agreeing with the results of in vivo CAM, the findings in the EA.hy926 cells once again indicated the ability of sesamin on hindering the pathological angiogenesis. This study endorsed the findings of Tsai et al. stating that sesamin can inhibit the VEGFA-induced proliferation and migration of HUVECs endothelial cells. However, sesamin was claimed to inhibit the proliferation and migration of uninduced HUVEC as well in that study, contradicting our results [20]. The different endothelial cell types may respond in a different way to sesamin, accounting for the distinct results.

The responsible signal transductions for the inhibitory effect of sesamin on VEGFA mediated angiogenesis were also investigated. We focused on the activation of p38, ERK, PLCγ1, Src, and FAK pathways, given that activation of ERK through PLCγ1 was reported to associate with the regulation of endothelial cell survival and proliferation while that of p38 and FAK was to control the migration of endothelial cells and that of Src was to regulate cell-cell contacts, proliferation, and migration [7,32]. Although the study by Byung-Hee Chung showed that 30 μM of sesamin could induce the activation of ERK, Akt, and FAK in uninduced HUVECs [19], we did not find the activation of these pathways by sesamin in EA.hy926. In contrary, VEGFA obviously induced all signaling pathways related to the activation of angiogenesis and sesamin pretreatment significantly impeded the activation of Src and FAK by VEGFA but not that of ERK, p38, and PLCγ1. Previous studies in HUVECs reported that total saponins and carvedilol could inhibit VEGFA-induced angiogenesis by the inhibition of FAK and Src activation, respectively [30,33], implying that Src and FAK signaling might be responsible for the proliferation and migration abilities of endothelial cells. The results of this study and previous studies altogether indicated the role of Src and FAK pathways in mediating the inhibitory effect of sesamin on angiogenesis under high VEGFA conditions. 

As mentioned above, endothelial cells in the form of tip or stalks cells work together in a harmonious way in sprouting angiogenesis. While the spear head tip cells migrate and lead the sprout towards the stimulant, a high VEGF gradient, they suppress the tip cell phenotype in the adjacent cells, which then become stalk cells. Stalk cells highly proliferate and facilitate the guiding of tip cells, resulting in elongation of the new vessels. Notch signaling pathway, which plays a critical role in cell fate determination, is involved in the specification of endothelial cells into tip and stalk cells [9]. When VEGF interacts with its main receptor VEGFR2, also known as KDR, the expression of the Dll4 ligand is upregulated in tip cells, which in turn activates Notch signaling in the neighboring stalk cells, resulting in increased proliferation but suppression of tip cell-behavior in these cells (lateral inhibition). Notch activation also reduces VEGFR2 expression in the stalk cells. In contrast, VEGFR2 expression is increased in tip cells as they have low activation of Notch signaling [5,9]; consequently, tip and stalk cells exhibit distinctive gene expression profiles. In this study, we studied the expression of angiogenic genes in VEGFA-induced EA.hy926 cells and the effect of sesamin on them. The results showed that when cells were induced with VEGFA, the *NOTCH1* expression was significantly induced, whereas *Dll4*, and *VEGF* expressions were not significantly changed. *KDR* expression was reduced, though not significant, by VEGFA treatment. This profile of high Notch and low KDR expressions proposed that endothelial cells in this study represent stalk cells, although it cannot be definitely distinguished since we used monolayer endothelial cell line lacking 3D interaction. Interestingly, sesamin was found to reduce the Notch1 induction by VEGFA significantly in both gene and protein levels. Previous studies found that inhibition of Notch signaling leads to an increased formation of non-functional vascular sprouts in tumor vasculature, which was explained by the de-repression of tip-cell behavior in endothelial cells [34]. Since sesamin was shown to reduce vascular branching in the previous experiment using the CAM model, the finding of Notch expression inhibition by sesamin was found to be contradicting. However, Liu et al. reported that vascular network formation was partially inhibited by blocking Notch signaling on the other hand [28]. These conflicting results might be explained by the plausible mechanism: inhibition of Notch 1 signaling will hinder the proliferation of stalk cells, a necessity for the elongation of new sprouts in angiogenesis. The results of inhibition of VEGFA-induced proliferation and migration of EA.hy926 cells also support this explanation. 

Activation of alternative angiogenic signaling pathways is contributing to the resistance against VEGF blockade therapy in cancer patients. Angiopoietin 2 (Ang2), being the antagonist of Ang1 that stabilizes nascent vessels by recruiting mural cells, is implicated in the formation of unstable and leakier vessels and it is upregulated in many cancers [13]. Hence, we examined the effect of sesamin on this pathway; specifically the expressions of *Ang1*, *Ang2,* and their receptor *Tie2*, in EA.hy926 cells. Nevertheless, no conclusive finding was obtained. Sesamin increased the expression of *Ang1* under VEGF influence of 12 h-duration, suggesting its potential to help the newly formed vessels to become stabilized, but the finding was not confirmed as the effect was not significant and disappeared in the 24-h period. On the other hand, treatment with sesamin alone reduced the expression of *Ang1* and increased that of *Ang2* in EA.hy926 cells without VEGF induction, although it was not significant. Therefore, the effect of sesamin on angiopoietins is yet to be confirmed in future studies using other endothelial cell types or a different dose and duration of the sesamin treatment.

## 5. Conclusions

Summarizing our study, sesamin inhibited in vitro and in vivo VEGFA-induced angiogenesis, while there was no influence on angiogenesis without induction, and this effect was accounted for by the reduction of the activation of Src and FAK induced by VEGFA. In addition, inhibition of Notch signaling, which was crucial for the proliferation of stalk cells, may also be responsible for the inhibitory effect of sesamin on VEGFA-induced angiogenesis. These results altogether highlighted the potential of sesamin as an anti-angiogenic therapeutic adjuvant, nonetheless, confirming that its effect in therapeutic animal models for accessing functions of the vasculatures is an absolute must.

## Figures and Tables

**Table 1:** The real-time PCR primer sequences.

Gene	Real-Time PCR Primer Sequence (5′-3′)	Reference
*Ang1*	Forward: 5′ CAGGAGGATGGTGGTTTGATG 3′ Reverse: 5′ TGGTTTTGTCCCGCAGTATAGAA 3′	NM_001146.4
*Ang2*	Forward: 5′ AGCTGTGATCTTGTCTTGGC 3′ Reverse: 5′ GTTCAAGTCTCGTGGTCTGA 3′	NM_001118887.1
*Tie 2*	Forward: 5′ GATTTTGGATTGTCCCGAGGTCAAG 3′ Reverse: 5′ CACCAATATCTGGGCAAATGATGG 3′	NM_000459.4
*VEGFA*	Forward: 5′ CTACCTCCACCATGCCAAGT 3′ Reverse: 5′ AGCTGCGCTGATAGACATCC 3′	NM_001025366.2
*KDR*	Forward: 5′ AGCATGGAAGAGGATTCTGG 3′ Reverse: 5′ CGGCTCTTTCGCTTACTGTT 3′	NM_002253.2
*NOTCH1*	Forward: 5′ GTCAACGCCGTAGATGACC 3′ Reverse: 5′ TTGTTAGCCCCGTTCTTCAG 3′	NM_017617.5
*Dll4*	Forward: 5′ GCACTCCCTGGCAATGTACT 3′ Reverse: 5′ CGACAGGTGCAGGTGTAGC 3′	NM_019074.3
*GAPDH*	Forward: 5′ CCCTTCATTGACCTCAACTA 3′ Reverse: 5′ AGATGATGACCCTTTTGGCT 3′	NM_001289745.1

## Data Availability

Not applicable.

## Supplementary Materials

The following supporting information can be downloaded at: https://www.mdpi.com/article/10.3390/biomedicines11010188/s1, Table S1: Normalized gene expression (ΔCт) of each target gene against GAPDH gene.

## Abbreviations

Dll4, Delta-like4; VEGF, Vascular endothelial growth factor; ANG, angiopoietin; *KDR*, Kinase insert domain receptor; SE, sesamin; CAM, Chick Chorioallantoic Membrane.
