# Supplementary material for: Sesamin Attenuates VEGFA-Induced Angiogenesis via Inhibition of Src and FAK Signaling in Chick Chorioallantoic Membrane Model and Human Endothelial EA.hy926 Cells
Source: Biomedicines. 2023 Jan 11;11(1):188. doi: 10.3390/biomedicines11010188 (PMC9855418; doi:10.3390/biomedicines11010188)
Supplement: Supplementary file 1 [file biomedicines-11-00188-s001.zip › biomedicines-2081214-supplementary.pdf]

Supplementary information of Real time RT-PCR

Table S1: Normalized gene expression (ΔCт) of each target gene against GAPDH gene

Target Gene

**NOTCH1**		normalized gene expression (ΔCт)		
		Round 1	Round 2	Round 3
12 hr.	Control	9.01	9.10	8.48
	VEGF	8.62	9.22	8.67
	VEGF+SE 3 μM	8.65	8.21	7.65
	VEGF+SE 5 μM	9.21	8.97	4.88
	VEGF+SE 10 μM	8.74	9.05	9.00
	SE 10 μM	8.54	8.84	8.72
24 hr.	Control	8.72	6.94	8.28
	VEGF	6.94	6.68	7.30
	VEGF+SE 3 μM	9.04	8.41	9.03
	VEGF+SE 5 μM	8.41	8.05	8.55
	VEGF+SE 10 μM	9.15	8.83	8.91
	SE 10 μM	9.15	8.96	8.81

**Dll4**		normalized gene expression (ΔCт)		
		Round 1	Round 2	Round 3
12 hr.	Control	10.67	10.78	10.32
	VEGF	10.05	10.62	10.81
	VEGF+SE 3 μM	10.17	10.73	10.55
	VEGF+SE 5 μM	10.49	10.75	10.23
	VEGF+SE 10 μM	9.93	10.48	10.15
	SE 10 μM	10.34	10.49	10.54
24 hr.	Control	9.70	10.36	10.59
	VEGF	9.48	10.13	10.38
	VEGF+SE 3 μM	9.48	10.33	10.39
	VEGF+SE 5 μM	9.09	10.22	10.04
	VEGF+SE 10 μM	9.41	10.55	10.06
	SE 10 μM	11.25	10.77	11.01

**VEGF**		normalized gene expression (ΔCт)		
		Round 1	Round 2	Round 3
12 hr.	Control	8.78	7.73	8.63
	VEGF	9.03	9.03	9.20
	VEGF+SE 3 μM	9.45	8.32	9.28
	VEGF+SE 5 μM	9.21	8.47	9.30
	VEGF+SE 10 μM	9.41	9.06	9.07
	SE 10 μM	9.55	8.26	9.45
24 hr.	Control	8.61	8.78	8.82
	VEGF	9.24	7.85	8.86
	VEGF+SE 3 μM	8.79	8.51	9.00
	VEGF+SE 5 μM	8.86	8.73	8.82
	VEGF+SE 10 μM	8.84	8.57	9.10
	SE 10 μM	8.85	8.85	8.74

**KDR**		normalized gene expression (ΔCт)		
		Round 1	Round 2	Round 3
12 hr.	Control	8.15	8.21	7.92
	VEGF	8.26	8.10	7.11
	VEGF+SE 3 µM	7.95	8.16	7.39
	VEGF+SE 5 µM	8.54	7.85	7.41
	VEGF+SE 10 µM	8.35	7.69	6.84
	SE 10 µM	8.23	7.67	7.48
24 hr.	Control	7.68	7.28	6.71
	VEGF	7.96	7.66	6.51
	VEGF+SE 3 µM	7.96	7.66	6.97
	VEGF+SE 5 µM	7.89	7.29	6.50
	VEGF+SE 10 µM	7.90	7.46	6.85
	SE 10 µM	8.53	7.82	7.37

**ANG1**		normalized gene expression (ΔCт)		
		Round 1	Round 2	Round 3
12 hr.	Control	6.33	6.04	6.66
	VEGF	6.27	5.87	6.50
	VEGF+SE 3 µM	6.15	5.80	6.19
	VEGF+SE 5 µM	6.06	5.65	6.93
	VEGF+SE 10 µM	5.76	5.36	6.70
	SE 10 µM	6.34	6.11	6.62
24 hr.	Control	5.38	5.21	6.33
	VEGF	5.35	5.13	6.52
	VEGF+SE 3 µM	6.15	5.76	6.51
	VEGF+SE 5 µM	5.93	5.59	5.58
	VEGF+SE 10 µM	6.03	5.68	7.26
	SE 10 µM	6.26	5.79	6.64

**ANG2**		normalized gene expression (ΔCт)		
		Round 1	Round 2	Round 3
12 hr.	Control	10.90	11.30	11.05
	VEGF	11.00	10.89	10.68
	VEGF+SE 3 µM	11.18	10.71	10.04
	VEGF+SE 5 µM	10.72	10.83	10.32
	VEGF+SE 10 µM	10.86	10.65	10.26
	SE 10 µM	11.06	10.53	10.80
24 hr.	Control	10.85	10.52	11.15
	VEGF	10.48	10.42	10.97
	VEGF+SE 3 µM	10.49	10.37	10.40
	VEGF+SE 5 µM	10.85	10.44	10.06
	VEGF+SE 10 µM	10.49	10.08	10.79
	SE 10 µM	10.53	10.23	10.85

Tie2		normalized gene expression (ΔCт)		
		Round 1	Round 2	Round 3
12 hr.	Control	4.82	4.08	3.08
	VEGF	4.71	2.52	2.95
	VEGF+SE 3 µM	4.50	3.23	3.01
	VEGF+SE 5 µM	4.29	3.14	2.51
	VEGF+SE 10 µM	4.23	2.47	2.07
	SE 10 µM	4.27	3.13	2.54
24 hr.	Control	4.49	3.29	3.11
	VEGF	4.33	2.46	2.48
	VEGF+SE 3 µM	4.08	2.64	2.47
	VEGF+SE 5 µM	3.52	1.64	1.21
	VEGF+SE 10 µM	4.03	2.55	3.12
	SE 10 µM	4.43	3.23	2.46
